# Supplementary figures and images for: Brain dynamics for confidence-weighted learning
Source: PLoS Comput Biol. 2020 Jun 2;16(6):e1007935. doi: 10.1371/journal.pcbi.1007935 (PMC7292419; doi:10.1371/journal.pcbi.1007935)

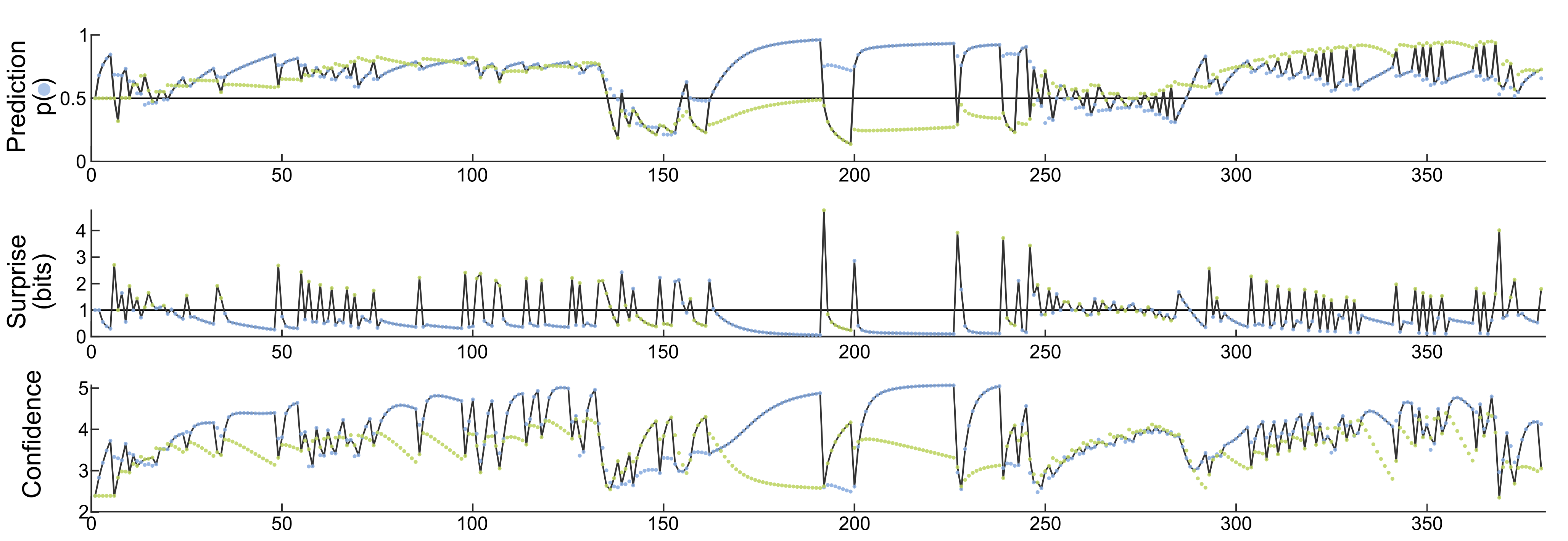

Supplement: S1 Fig — We consider an example sequence in which observations are color-coded (blue and green). The top graph shows the posterior inference of transition probabilities made by the Bayes optimal model in the course of sequence presentation: the green dots show the probability for the next item to be blue if the previous item was green, the blue dots show the probability for the next item to be blue if the previous item was blue. The black line shows the prediction conditioned on the identity of the item previously presented. The middle graph shows the surprise (in bits) corresponding to the actual observations, whose identity on each trial is color coded; the sequence of colored dots therefore represents the sequence of observations. The bottom graph shows the Bayes optimal confidence (i.e. posterior precision) associated with the inferred transition probabilities, using the same convention as in the top graph. Several aspects are noteworthy. First, both surprise and confidence show marked dynamics within the course of an experimental session (380 stimuli). Second, those two dynamics are distinct, for instance, surprise may be rather steady while confidence changes (e.g. from stimulus 250 to 280). Third, predictions and the associated confidence can change repeatedly from trial-to-trial when transition probabilities differ, e.g. from stimulus 300 to 340. Fourth, similar predictions can be accompanied by different confidence levels (e.g. from stimulus 100 to 140, both transition probabilities support a prediction around 0.75 and yet, the prediction is associated with higher confidence when the previous observation is blue). (TIF) [file pcbi.1007935.s001.tif]

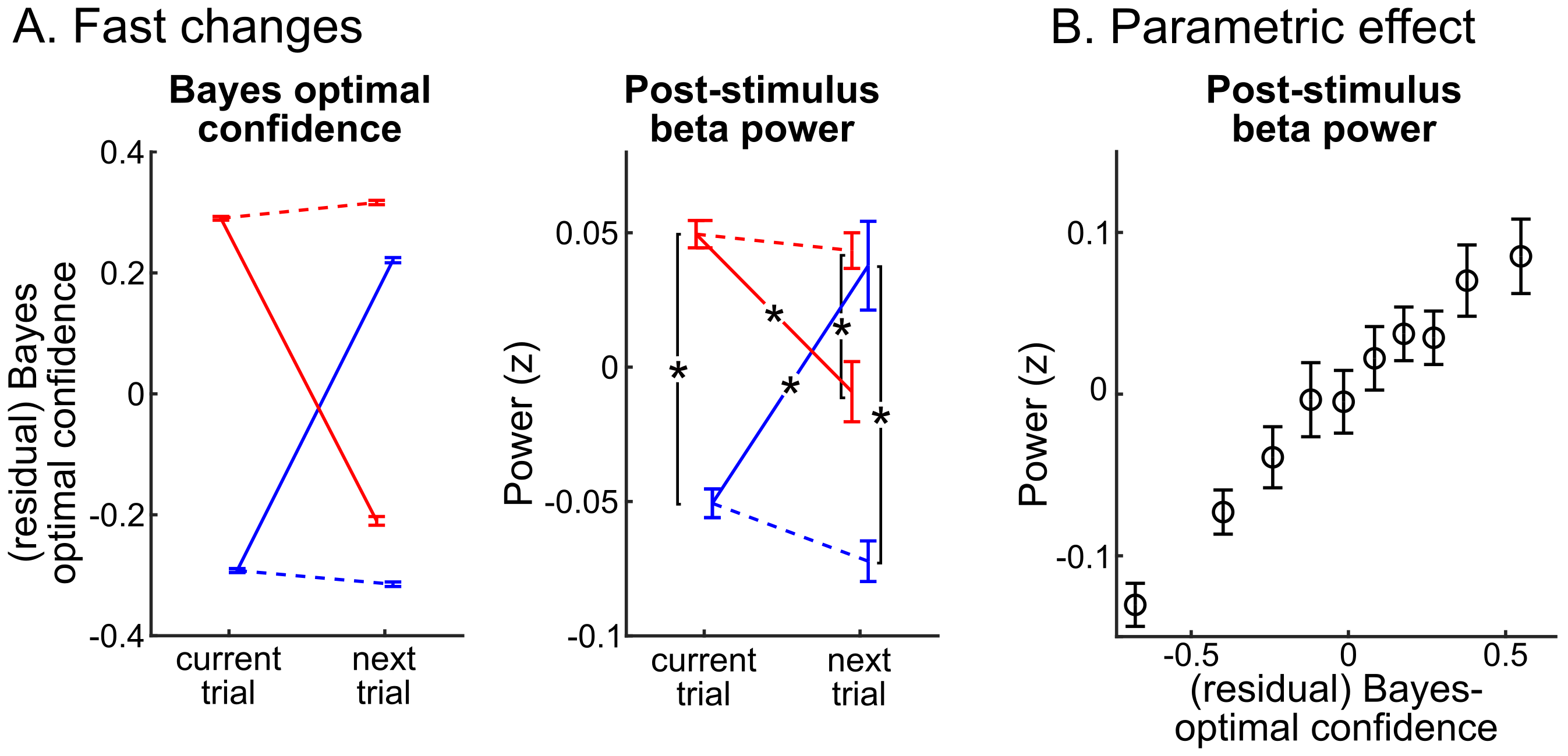

Supplement: S2 Fig — (A) Pairs of adjacent trials were sorted into high and low (red vs. blue) residual Bayes-optimal confidence on the current trial, and further sorted into high and low residual Bayes-optimal confidence on the next trial, therefore forming pairs that kept similar levels (dashed line) or changed drastically (plain line). The (z-scored) power in the post-stimulus beta-band cluster (Fig 3A) showed fast, trial-to-trial changes that parallel the residual Bayes-optimal confidence (*: p<0.005, paired t-test). (B) The correlation between post-stimulus beta-band power and (residual) Bayes-optimal confidence was not driven by specific values but indeed corresponds to parametric changes. Error-bar: s.e.m. (TIF) [file pcbi.1007935.s002.tif]

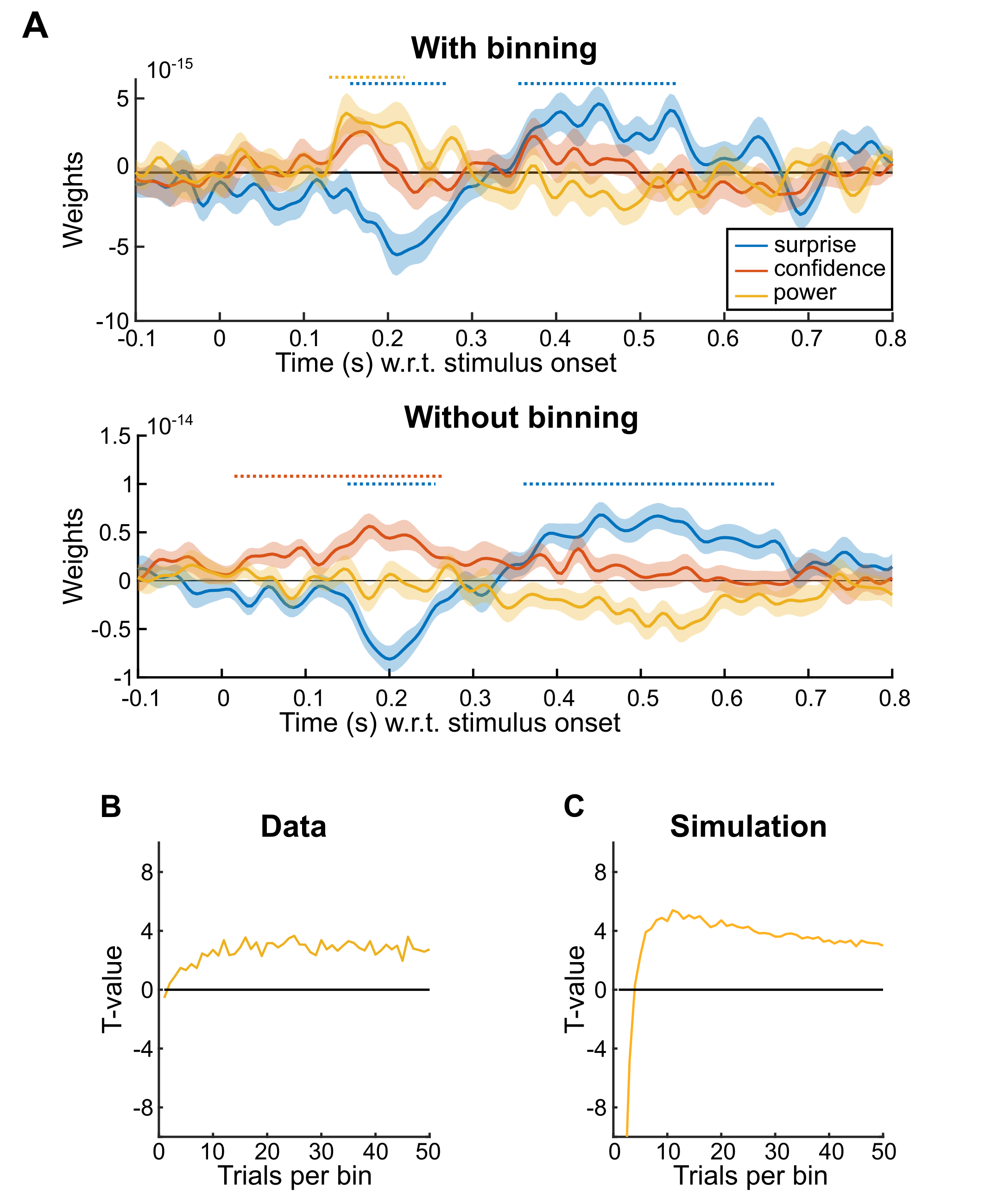

Supplement: S3 Fig — (A). The top panel is the same as Fig 5B. For this analysis, 10 consecutive trials were averaged into bins prior to estimating the regression. The bottom panel shows the results when trials are not binned (or equivalently, when there is only one trial per bin). Note the selective change around 200 ms for the effect of power. (B) explores this regression analysis specifically around 200 ms (averaging across significant time points, Fig 2A middle) and shows the significance (group-level t-value) of the effect of post-stimulus beta-band power onto to the ERF, depending on the number of consecutive trials per bin. (C) is the same analysis as in (B) but for signals simulated as follows: POWERneural = β1*CONF + ηPOWER, ERFneural = -(β2*SURP -β3*POWERneural) + εERF, POWERmeas. = POWERneural + εcommon; ERFmeas. = ERFneural + εcommon. CONF and SURP are the Bayes-optimal surprise and confidence, POWERneural and ERFneural are the true beta-band power and evoked response, POWERmeas. and ERFmeas. are the signals measured by MEG; ηPOWER is auto-correlated Gaussian noise, εERF and εcommon are identically distributed Gaussian noises. For the simulation, ηPOWER has SD = 1 and auto-correlation ρ = 0.5; εERF and εcommon have SD = 1, ρ = 0; β1 = 0.25; β2 = 0.25, β3 = 0.5. Those parameters are arbitrary, not fit to the data. (TIF) [file pcbi.1007935.s003.tif]
