# Supplementary material for: Brain dynamics for confidence-weighted learning
Source: PLoS Comput Biol. 2020 Jun 2;16(6):e1007935. doi: 10.1371/journal.pcbi.1007935 (PMC7292419; doi:10.1371/journal.pcbi.1007935)
Supplement: Attachment — Submitted filename: ResponseToReviewers.pdf [file pcbi.1007935.s004.pdf]

I thank the reviewers for their supportive comments and suggestions which helped me to improve the proposed article. Here is a summary of the main actions in the revised version:

- I added a new section about behavioral results, providing new details on the relationship between subjects' data and the the Bayes optimal model vs alternative models.
- I added the source localization of the effect of confidence on beta band power
- I investigate the model update more quantitatively
- I changed figures (now same time window for evoked responses; a different way of splitting the data to illustrate the results)
- I more clearly defined key notions in the introduction and simplified the terminology
- I amended the discussion with more references and cautionary notes

Below, you will find of a point-by-point response.

NB: the line numbers below refer to the PDF manuscript with tracked changes.

Reviewer #1

In this paper the author examines behavioural and neural correlates of optimal inference during a probabilistic learning task. In each trial, participants hear one out of two possible tones (A or B). In some trials participants are asked to predict the probability with which the tone will be repeated on the subsequent trial as well as their confidence associated to this probability estimate. During the experiment, the generative probabilities (p(A|A) and p(B|B)) can change at random points with very low probability. The task structure is transparent to the participants. Behaviour in the task approximates, to some extent, Bayes-optimal inference. Additionally, MEG data show signatures of optimal inference, in the sense that the neural activity in sensors representing surprise (or likelihood) is also modulated by bayesian confidence. This co-modulation of neural activity by the likelihood and confidence is consistent with Bayesian updating where likelihoods are upweighted when the variance of the prior is broad (i.e. low confidence). Further analyses show that brain state, indexed by pupil-related arousal and beta-band activity, covaries with confidence.

Overall the research presented in this paper is carefully done and thought of, following on the author's previous work using the same probabilistic task and Bayesian framework. I have the following comments.

The behaviour of the participants in this paper is not analysed in depth. In Figure 1 D-E, some descriptive results are presented in relation to the predictions of the Bayes-Optimal model. Additionally, the probability estimates and confidence reports of the participants correlate, to some extent, with those of the bayesian model. These are the only points in which the relationship between the optimal model and behaviour is dealt with. I do not see these points as conferring evidence for or against the optimal model. In fact, the presented results are not informative. One for example could devise a simpler heuristic model, which could exhibit the same correlations with the bayesian estimates and confidence levels. The author is careful about this aspect, mentioning at several points that the bayesian model offers a formalisation of the learning process at the computational level. However, in light of the MEG data presented later, I am not sure if a computational level model —that presumably predicts broad features of behaviour but also misses on key algorithmic aspects of the learning process— is appropriate. Of course, if the optimal model was shown to explain the data better relative to other models the subsequent MEG results would bear larger importance. Below I provide a few recommendations on how to further scrutinise behaviour:

(a) It has been shown that people exhibit several biases when perceiving streams of data (e.g. hot hand fallacy, ignoring rare events etc) that are inconsistent with the optimal model. Are subjective probability and confidence estimates influenced by irrelevant features, for instance the fraction of alternations in the past 10 trials (keeping the counts for A and B fixed)?
(b) How well and how much faster (or slower) do people respond to change points relative to the optimal model?
(c) In intervals where the transition probabilities have larger entropies, do people report overall lower confidence?
(d) How correlated are reported probability estimates and reported confidence levels in the data and in the optimal model?

The above are just starting points towards falsifying (or failing to do so) the optimal model. I appreciate that in the absence of overt behaviour in most of the trials this point may be difficult to address. However, at least one simpler model should be compared both qualitatively and quantitatively to the optimal model.

The distinction between the computational and algorithmic level pointed by the reviewer is interesting. In the present study, I make no claim about how the brain may derive those probability estimates and the associated confidence levels (which is the algorithm). **I see the algorithmic description as a future step**, and in this sense, the current results are perhaps interesting because they list some properties of the human inference that such algorithm should exhibit.

I should also clarify that I don't mean that subjects compute the optimal model. Such claims are invariably wrong since deviations from optimality always exist. That being said, the optimal solution remains a goal for the brain, since it endows the learner with optimal estimates; it is thus an informative benchmark: to what extent do subjects approximate the optimum? **I amended the discussion (L. 420-424) to mention those two points (algorithm and optimality).**

The extent to which subjects approximate the optimal solution can be characterized in term of correlation between subjects' data and the optimal values (Fig. 1 D-E), and also by testing different normative properties of confidence reports. Listing such properties was the goal of my 2015 paper in Plos Comp Biol. **I initially omitted a detailed report of those properties for brevity, and I put it back in the revised version (L. 156-179)**. It nicely replicates the 2015 results, and motivates the use of the normative model. Those properties could also be seen as a set of heuristics for reporting sensible confidence levels during the task. However, **subjective confidence is more sophisticated than those heuristics here since it still covaries with optimal confidence once the contribution of those heuristics have been linearly regressed out (L. 180-189)**.

More specifically, points (a) and (b) are difficult to address due to the quite occasional nature of the questions asked to subjects. Point (c) is part of the several normative properties tested (L. 162-166). Point (d) is also now reported (L. 159-161): there is no linear correlation between probability estimates and confidence.

2) The MEG analysis presented in Figure 2 shows that key quantities in the optimal model correlate with the evoked MEG activity. The validity of this result is directly dependent on the unresolved point above (how well does the optimal model describe the learning process). For instance, bayesian confidence and counter_A*counter_B (with counter_X keeping track of how many times X tone occurred in the last N trials in a suboptimal learning model) can be two strongly correlated quantities. Therefore the mere occurrence of these neural signals per se does not provide further evidence for the optimal model. The author here focuses on a subset of sensors on a specific time interval, which are jointly modulated by confidence and surprise, with opposite signs as prescribed by the optimal model. The rationale of this approach should be further motivated and validated. Wouldn't it make also sense to show the activity of the "surprise" sensors as a function of high and low confidence? The encoding of confidence and surprise seems to be very variable: it appears lateralised (why?) and for the surprise metric there is a sign flip after 0.22 seconds, which I guess reflects the return of the signal back to baseline. All these aspects (the sign of encoding) appear to be arbitrary especially given the fact that overt behaviour is not used in any way to constraint this analysis (akin to the way beta band activity later on is used to predict subjective confidence; why is this not done for the sensor level data?).

Some of the points above (question 1) are also relevant here.

Plot 2-B showing the data for a sub-set of sensors is essentially for illustrative purpose, the key analysis is the one with all time points and sensors with correction for multiple comparisons. I now stress it (L. 215).

Regarding the lateralization of the effect, I don't have a good interpretation of it. I simply note that the effect of surprise is also a bit lateralized (see insets corresponding to 0.09-0.14s and 0.4-0.6s in Fig 1A).

The interpretation of the sign of the signal is tricky with MEG (or similarly EEG) due to a baseline effect and the evolving distribution of sources. The surprise effect shows opposite signs around 200 ms and after 400 ms, which is consistent with the the topography of the effect presented in Fig 2: on the anterior, left sensors (shown in Fig 2B), the effect is negative (200 ms) and then positive (>400 ms), with different topographies, suggesting a different configuration of the underlying sources.

The reviewer points that contrary to time-frequency data, the evoked responses are not compared to behavior. This is because the evoked responses are evoked by the stimulus, which may be more or less surprising. During question trials, there is only the question, no stimulus, and thus no stimulus-evoked responses. By contrast, some time-frequency correlates reported here precede the stimulus, therefore, they can be measured even when the stimulus is omitted and replaced by a question, offering the possibility to compare the "pre-

stimulus" effect (when actually not stimulus is presented) with the actual confidence reported by the subject. **I now stress this difference (L. 312-314).**

3) The time-frequency analyses (Fig. 3) are presented as reflecting brain state fluctuations. It is not clear why for instance beta-band power here is related to arousal and is not evoked, related to task events. Additionally, in this section the authors speaks about arousal but does not clarify the direction of the relationship between confidence and arousal. It seems that higher confidence is associated with larger arousal (I inferred this indirectly). How can this effect be rationalised given also the fact that higher confidence is associated with evidence downweighting? Finally, the causality relation between arousal and confidence here is not determined. The author should discuss this limitation.
In the introduction, **I now more clearly define the notion of "brain state"** see L. 87-91, and stress in particular that it is in part under top-down control. The results indeed indicate that both beta-band power and tonic pupil size change as a function of optimal confidence, which depend on the presented sequence rather than being a participant-specific feature.
I now write explicitly in the text that "lower confidence was associated with larger tonic pupil size (increased arousal)" see L. 337, and the negative weight in the regression analysis (Fig 4C). This effect, accompanied by a reduction of the surprise response (Fig 5 C), is consistent with a down-weighting of sensory evidence found in previous studies (see discussion, L. 470-480).
The discussion now includes a cautionary note on causality: only correlation are tested in the current study, see L. 526-528.

Minor comments:

4 — What does the residual bayesian confidence correspond to? I believe that if no change points could occur the bayesian confidence could be perfectly predicted by the regressors used here. However, it will be useful to gain an intuition of this residual confidence and what it captures.
I now discuss more this metric when first introduced, see L. 201-209. These are the residuals once the linear and non-linear (square, log) effects of the probability estimate have been regressed out. For instance, confidence tends to increase for more extreme probabilities (closer to 0 or 1), which is no longer the case when considering the *residual confidence*.
If there were no change point, the estimated probability would converge (to the generative probability) after some time; therefore, the estimated probability would remain constant after some time. By contrast, confidence (and residual confidence) would continue to increase.

5 — In Figure 2B statistics should be shown. Also there are four bins based (I believe) on independent median splits on confidence and surprise. It would be more informative if these bins where constructed in a conjunctive fashion (e.g. low confidence & low surprise).
The plot is for illustrative purpose only, I would prefer not to report stats, because this statistical test would not be independent from the sensor selection: it would be "double dipping". Fig 2B reports the signal in sensors showing an effect of confidence (sensor selected based on the analysis in Fig 2A), which overlap with those showing an effect of surprise. By selection, effects of surprise and confidence are expected.
Regarding the split please see R3-10. There are pros and cons in principle. The independent median splits ensure that the same cut-offs are used for all categories but the number of trials may differ; the conjunctive fashion ensures an equal number of trials per category, but then the cut-offs differ and one must choose whether to split first based on confidence or surprise. In practice, **they happen to be quite similar**: if I focus on sensors and time point corresponding to the confidence cluster (it is around 200 ms), for which I want to illustrate the ordering of conditions predicted by the (optimal) confidence-weighting of surprise, we get:

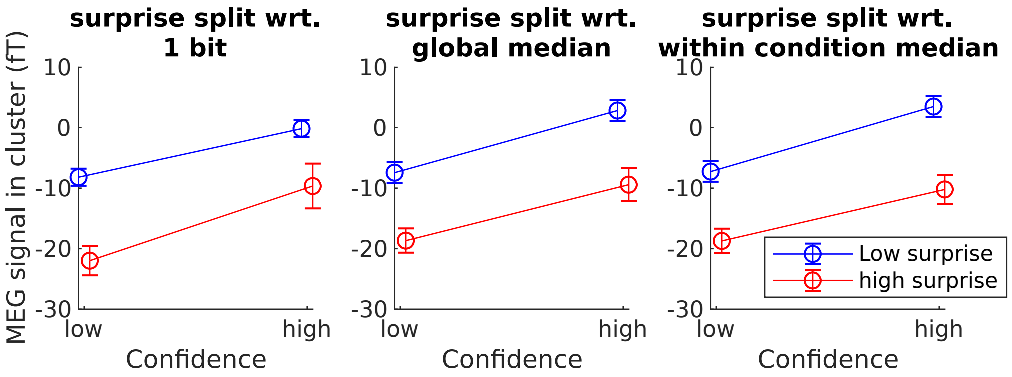

Using a "global" median split for confidence and surprise separately, or split by confidence first, and then by surprise returns almost the same values because the splitting values are extremely similar: median surprise is 0.7518±0.0651 across all trials, and 0.7523±0.0674 and 0.7556±0.0747 within low and high confidence trials respectively (mean±SD across participants).

6 — The analyses in Figure 5 is based on running two regression models. Given that the tonic pupil and beta power are not correlated why not run a single regression model?
Tonic pupil size and beta power are not correlated (i.e. $\rho \approx 0$), but they both correlate with (optimal) confidence. When adding all variables in the same regression, there is thus (partial) colinearity between optimal confidence, tonic pupil, beta power. This is why I decided to report distinct regression models in Fig. 5. However, note that **the single regression model is also reported in the text, see L. 390-396**. Another reason to report the two regression models is because due to an unfortunate technical problem, pupil size is available only for 18 participants; analyses including pupil size thus have a reduced statistical power compared to the other analyses. Fig 5 shows the effect of beta power in all (24) subjects, whereas the effect of pupil size (and the effect of pupil size and beta power from a single regression model) correspond to 18 participants.

7 — The writing is overall good but I had to resort to the author's previous papers in order to clarify what each of the central metrics corresponds to (surprise, prediction, predictability etc). A longer introduction in which these key quantities are unpacked will be useful.
Thank you for this suggestion, and the time spent looking at the previous papers. **I amended the introduction in order to have a standalone description**, see L. 66-79.

Reviewer #2
The general aim of Meyniel was to investigate whether confidence - formalized in terms of the precision of posterior predictive distributions of a Bayesian optimal model - modulates neural responses (MEG signal) evoked by surprising events during a learning task. A second and interrelated aim was to see whether confidence is associated with brain states (spectral power or pupil-linked arousal) and in turn, whether such confidence-weighted brain states are associated with surprise-evoked responses. The author found that confidence correlated with some evoked responses that overlapped in topography and time some responses associated with stimulus surprise (measured in terms of Shannon's surprisal). Moreover, confidence was also associated with some brain states, namely, with tonic pupil size and with both pre- and post-stimulus onset alpha and beta power. These brain states were in turn associated with a surprise-evoked MEG signal response.
This is a very interesting and overall valuable study, and I think it could make an important contribution to contemporary literature on the predictive brain. To maximize its contribution, however, I think some conceptual and methodological issues should be addressed in a revision. I list these issues in the order in which they occurred to me.

Major comments:

1. The author was mainly interested in explore how confidence modulate surprise responses, an effect that is captured by their interaction. However, he states that their effect is theoretically mostly additive (lines 191-192). I think it is important to further argument this point: why is it theoretically mostly additive? Any

reference? Why should the absence of an interaction not be considered a limit on the conclusion that surprise responses are dampened for higher confidence?
This is an important point indeed. In this paper, I am interested in the confidence-weighting of surprise inasmuch it is key to update the estimate optimally. This update can be formalized; the Kullback-Leibler divergence between prior and updated distributions is often used to this end (e.g. O'Reilly et al 2013, quoted). Here, the update (KL divergence) happens to be captured to a large extent as an additive effect of surprise and confidence (when defined as they are here). Looking for an update signal by means of linear regression therefore boils down to including surprise and confidence separately. **I amended the results see L. 239-243 and added a new paragraph in the methods to explain this point, see L. 677-689**.

2. The effect of confidence on MEG evoked responses seems little and, indeed, it does not reach statistical significance in the analysis whose results are shown in Fig 5B. I think this aspect should be explicitly mentioned in the text since this effect is at the core of the first goal of the study.
The effect of confidence on evoked responses is not huge, agreed, but **the analyses are very conservative**:

- All regressions use the residual confidence, which removes linear and non-linear effects attributable to the stimulus probability (which contributes significantly to optimal confidence, see the new result section L. 162-166). When using raw confidence, the effect is bigger, for instance in Fig 2B, the confidence cluster spans 0.145 to 0.220 ms, 8 channels, and reaches a maximal t-value of 5.0 in the current analysis (with residual confidence). With raw confidence, the cluster spans 0.140 to 0.225 ms, 14 channels and reaches a maximum tvalue of 5.8.
- The statistical threshold is also stringent, corrected for multiple comparisons over a window of 900 ms in Fig 2A and Fig 5 (and also all sensors in Fig 2).
- In Fig 5, we add the beta-band power (or tonic pupil size) which correlates with confidence, and thus competes with the confidence regressor in the multiple regression analyses reported.

This being said, **I don't want to undermine the weakness pointed by the reviewer, I explicitly acknowledge it in the revised discussion** (see L. 495-497).

3. Previous studies have shown that behavioral and neural responses associated with the amount of surprise of a stimulus can be distinguished from responses associated with how much the internal model is updated (e.g., O'Reilly et al. PNAS 2013; Kobayashi & Hsu, JNeurosci 2017; Visalli et al., NeuroImage 2019). As also reported in the discussion, the precision of the posterior after trial t is also linked with how large is the divergence with the posterior at trial t+1. In other words, the precision is inversely related with the amount of updating (often measured using the Kullback-Leibler divergence). I was wondering why the author did not regress out also this updating measure in computing the corrected confidence (the Matlab code provided online also calculates the DKL). Moreover, I think that it would be interesting to see whether the confidence-modulated brain states are associated with or modulate possible DKL responses.
First, note that **the revised version now makes it clear that the model update can be quantified as KL divergence** (see L. 680-682). I also stress that **update (KL divergence) can be modeled as an additive effect of surprise and confidence** (see point R2-1).
The quoted papers resorted to different strategies to distinguish update from surprise:

- O'Reilly et al used a design in which a few surprising observations are irrelevant to update the current estimate because they explicitly (color cue) correspond to an unrelated generative process.
- Visalli et al 2019 use a similar idea, presenting also surprising samples that are explicitly irrelevant to update the current estimate. In addition, and contrary to my study, they explicitly instruct the subjects about change points positions. In my study, uncertainty about change points is a major source of lack of confidence.
- Kobayashi & Hsu 2017 distinguish (belief) update from surprise, by presenting the effect of update adjusted for surprise. Using the vocabulary of my study, the part of the update that remains after adjusting for surprise is actually confidence to a large extent.

In the current study, I am interested in confidence inasmuch it should in principle contribute to update the current belief. In that sense, correcting confidence for the update would remove the aspect of confidence that I am indeed interesting in.
Regarding the modulation of "update" responses (in the sense of KL divergence) by confidence-modulated brain states: this is actually the motivation behind Fig 5. I take the evoked response showing both an effect of confidence and surprise (isolated in Fig 2) as a putative update response, and test whether this evoked response changes as a function of confidence-modulated brain states (beta power and tonic pupil). **I spell out this point more clearly in the revised version** (see L. 355-359).

4. Linked to the previous point, in the discussion the author states that "Overall, the results indicate that changes in brain states indexed by beta-range power and neuromodulation, are related to differential responses to surprising stimuli, providing a mechanism for a dynamic confidence-weighting of learning". However such link with learning is indirect and not directly tested in the study. I think this fact should be addressed in the Discussion section.
This is quite true. **I added a new dedicated paragraph to stress this point in the discussion**, see L. 521-528.

5. Different critical alpha for both cluster formation and cluster-level correction were used from one analysis to another. Which was the reason? I think that such choices need to be justified.
I initially planned to do all analysis with alpha levels of 0.05 for cluster formation and correction. However, some effects appeared to be significant even when adopting more stringent thresholds, I thought the conclusion would appear more convincing if more conservative thresholds were used, e.g. 0.001 to form clusters (Fig 2). However, even with this more conservative threshold, some results remained overwhelmed by significant clusters, in particular the time-frequency analysis, and thereby more difficult to interpret and follow-up with further analyses. For the time-frequency data, I thus adopted a cluster-level correction of 0.01. For clarity, I report the alpha levels together with each analysis. **I amended the methods section to justify this choice, see L. 721-726**.

Minor comments:

6. In the methods I would better formalize and describe the regression models using formulas for each model and reporting the employed software. Moreover at lines 577-581, it is reported: "We often used the "residual" Bayes-optimal confidence as a predictor, which was computed by linearly regressing out the effect of prediction and predictability. More precisely, we estimated a multiple linear regression of the Bayes-optimal confidence, using as predictors the prediction itself (i.e. the Bayes-optimal estimate of the transition probability of the next stimulus, p(A)), its square and logarithm, log(p(A)) and log(p(B))". However, predictability was defined at line 128 as the entropy of the prediction. What do square and log of p(A) refer to?
Regarding the regression models: instead of writing formulas in the methods, I prefer to provide the list of predictor variables corresponding to each regression analysis directly in the text. **I have screened the text to ensure I do it exhaustively. I also added the reference to the software**, see L. 670-671.

The entropy of the prediction, H=-(p(A)log(p(A) + p(B)log(p(B)), corresponds to an inverted U-shape function of the prediction. Confidence also follows, to some extent, the prediction following a U shape. I could have computed the residual confidence as what remains when one linearly removes the effect of H only, however, I used a multiple regression with p, p^2, log(p) and log(1-p) in order to capture more flexibly any non-linear dependency between confidence and the prediction. **I revised several aspects of the text to clarify the notion of residual confidence, see L. 201-209 and L. 672-678. For simplicity, I also removed the notions "predictability" and "entropy of prediction" from the text**.

7. At line 152, the term "factor" is used to refer not only to the categorical variable (stimulus identity), but also to the continuous variables. I would use a more appropriate term, such as "predictor" or "regressor" in all the manuscript.
I replaced "factor" with "predictor" throughout the document.

8. At line 163, it is reported an effect of surprise between 80 and 155 ms, however, I thought that the analysis were run starting from 100 ms. Related to this point: why does the author not start the analysis from target onset? Which was the criterion to select the analysis time window. Why does the MEG signal analysis end at .8 s while the frequency analysis ends at .9 s?
The analysis was run from -0.1 to 0.8s, but an **unfortunate line break** split the hyphen away from 0.1, which must have caused the confusion. Thank you, I have discovered non-breakable hyphens in my text editor!
Your remark made me realize that despite the analysis was run from -0.1 to 0.8s, the Fig 2A showed data from -0.2 to 1s, therefore **I changed Fig 2A**, to match the -0.1 to 0.8s interval.

For the time-frequency analysis, unlike the evoked responses, I wanted to explore pre-stimulus signals, the window therefore starts at -0.5 (instead of -0.5). I could have stopped it at 0.8s as well, it was really arbitrary to go to 0.9s.

9. Linked to comment 2, the topoplots in Fig 2 have a colormap in the range [-3.5, 3.5] t-value. However, the max observed t has a value of ~10. It would be better to use different colormaps for each effect (or the same color map in the range [-max|T| max |T|] to be able to perceive the difference in effect size between those effects (this applies also to Fig. 3).
I have changed the topoplots. The new ones use [-max(|-T|), max(|-T|)]. The also now show the t-values of the data averaged within a time window, rather than the average of t-values.

10. Why in figs. 2B and C is surprise differently categorized in high and low?
In Fig 2C, I really wanted to have the same number of trials in each category to compute the contrast (this is often a concern for source localization). In Fig 2B I used a split with respect to 1 bit, to have expected vs. unexpected observations, which is imbalanced. I agree it may seem discrepant in the end, **the new figure 2B now uses a median split for surprise as well (as Fig 2B)**.
As shown in response to R #1-5, it does not change the conclusions.

11. At lines 207-208, the sentence "Power in low frequencies (<40 Hz) typically characterizes the state of large networks" needs references.
Done. It now appears L. 258.

12. In the Discussion (lines 431-432), it is reported that "It is noticeable that the modulation of evoked surprise responses was confined to early post- stimulus latencies (around 200 ms) rather than occurring later", however an effect of surprise was also found later.
To avoid confusion, **I rephrased**, the new sentence now reads as "it is noticeable that the modulation of the evoked responses *by confidence* was confined to..."

13. There is a typo in the right subpanel (topoplot) of Fig. 2A: [0.9 0.14]s instead of [0.09 0.14]s
Thank you! Corrected.

Reviewer #3
This manuscript reports data from an MEG study with healthy subjects who performed a perceptual learning auditory task, providing occasional behavioural responses pertaining to their sensory predictions and associated confidence. Pupil size was also monitored over time to test its relationship with confidence up-dating and surprise. The task used here is very close to the ones used by the author and collaborators in several previous studies. It is coupled here with Bayesian modelling and the analysis of various MEG components (evoked and oscillatory) at both the scalp and source level, to carefully assess the neural correlates of prediction, surprise and confidence, respectively. The main results that pertain to showing that the trial wise up-dating of confidence is well captured by Bayesian learning and can be related to evoked activity around 200ms, as well as beta power and pupil size, are of great interest to the field.
The manuscript is well structured and clearly written. Data analysis include several rigorous verifications that attest the robustness of the main findings. Although the manuscript would gain in readability if some of these additional analysis would be only shortly referred to in the main text and moved to an additional material section.
I started to rework the text following this suggestion, but I found it difficult to ensure a clear correspondence between main text and supplementary sections. Instead, **I decided to simply insert a new header "control analyses"** at the end of each section to serve as an umbrella for those details; the reader can now very easily skip those sections if not interested.

The discussion is quite thorough and put the present work in a useful perspective by relating it broadly to relevant recent studies pertaining to different domains (modelling, animal and human data, neurotransmitters and oscillations…).
This paper is suitable for publication and will be of interest to many. I only have minor suggestions which I hope will help further strengthening the manuscript.

General comments and questions

- How specific is this task? Subjects are indeed heavily trained and informed in details about the task hidden structure. Does it mean that the observed computations and the identified neural correlates are hardly generalizable?
The question about generalization arises whenever one presents the result of a single task. I prefer not to speculate on this, since I feel the question is really open and doing new experiments is the most valid way to answer it. I anticipate there are at least three aspects that could be relevant to discuss generality, and which could be changed in future experiments: 1) whether the subject knows about the true generative process of stimuli, 2) whether the task explicitly requires to compute a probability, 3) whether the subject pays attention to stimuli. **I added a new paragraph in the discussion to discuss generality**, see L. 515-520.

- Importantly, the crucial and related but different notions manipulated here such as prediction, predictability, surprise, prediction error should be more clearly and initially defined. If some of these terms are interchangeable, they could be mentioned once but then one notion should be referred to by a single term. Especially as the author rightfully insists on the importance and the difficulty in distinguishing between such notions like precision and predictability.
Thank you very much for pointing this. The **revised introduction now give a clearer definition of the main concepts** of interest (prediction as expected probability of stimulus; discrepancy between prediction and observation as surprise; confidence as the log-precision of the probability of stimulus), see L. 68-78. Most of the paper then uses only those few key notions. I only mention other concepts (e.g. prediction error) when discussing papers that refer to those concepts. I mostly removed the notion of predictability for clarity (only presented once), which I replaced with the notion of expected outcome uncertainty.

- Regarding source reconstruction: please clarify whether you computed the source of the difference (source of an effect) or the difference of the sources (the former yields more sensitive results). And regarding the interpretation of the qualitative findings, the author pin-points the implication of the right IFG and intermediate precentral sulcus (for surprise), however the observed topographies reveal a more spread network including TPJ and other regions, please complement or nuance your current report. Along the same vein, the sources pertaining to confidence do not pin-point towards a clear region or network. Please comment. And in relation to that, could you try to complement this source reconstruction by source localizing the effect of confidence on post-stimulus beta power?
I removed the specific indication about IFG and precentral sulcus to nuance my report, instead, I leave the exploration of the brain maps to the reader.
As suggested by the reviewer, **I included in the revised version the source reconstruction of the effect of confidence on post-stimulus beta power, see Fig 3B**.

- why not reporting the effect (or absence of effect) of prediction onto low frequency oscillations and pupil size?
Looking for neural manifestations of the prediction itself is very interesting. This is actually an analysis that we plan to run, although the task has not been designed to elicit easily decodable representation of the stimulus (the fact that there are effects of the stimulus identity on the stimulus-evoked response is nevertheless encouraging) and its potential modulation by confidence. However, the current study is focused on surprise (i.e. violation of prediction) and the confidence-weighting of surprise responses by confidence. The neural signatures of the prediction itself is an orthogonal question I think, **not in the scope of the present study**.

More specific and minor comments
- On figures 1.D, 1.E but also 3.D and S2.B, it is not clear to me how the data points were binned in order to build those figures
Fig 1D-E and 3-D use equally-filled bins of data. Fig 3.D uses bins of consecutive trials. **This is now clarified in the legend of Fig 1, 3 and S2**.

- lines 141 to 144: please unfold this argument which remains unclear to me.
I revised this section please see L. 144-151**.**

- Fig. 2B reports MEG evoked signals for conditions obtained by splitting data around the median. Since such a split is performed along both confidence and surprise, could you please report the number of trials that

fell into each of the four categories? Or clarify your strategy. Is it that after having split along one dimension, the data were further split along the other one? In which case it would be worth reporting the median values.
Please see R#1-5, **I revised this figure**. In the above response, I compare different ways of splitting the data. I do independent median splits and then use the conjunction, which happens to result in a number of trials which is essentially the same across conditions (333.1 to 334.5 on average across subjects).

- I found the use of the loose term 'Brain state' a bit disturbing. I would encourage the author to envisage reconsidering that use.
Thank you for raising this point. I used "brain state" as a shorthand and I agree that it was not properly introduced. **I revised the introduction to better define this notion up-front, see L. 87-91**.

- In the discussion, the part pertaining to related (MMN) findings using EEG, the author could also refer to:
Ostwald et al. Evidence for neural encoding of Bayesian surprise in human somatosensation. NeuroImage 62, 177–188 (2012).
Lecaignard et al. Implicit learning of predictable sound sequences modulates human brain responses at different levels of the auditory hierarchy. Frontiers in Human Neuroscience 9, (2015).
Thank you for pointing those relevant references; **I added them**, see L. 438, together with new references (Chennu et al 2013; Auksztulewicz & Friston 2015).

- Line 471: what you mean here is unclear to me.
I inserted an equation in the revised version to make this point clear, see L. 552.

- I noticed a couple of typos: Thank you!
end of line 411: noradrenaline; fixed.
line 500 (remove 'a': is given); fixed
Fig. S2 line 654 (Paris OF adjacent…); fixed
